# Implementation intentions to express gratitude increase daily time co-present with an intimate partner, and moderate effects of variation in *CD38*

**DOI:** 10.1038/s41598-022-15650-4

**Published:** 2022-07-09

**Authors:** Yen-Ping Chang, Baldwin M. Way, Paschal Sheeran, Laura E. Kurtz, Donald H. Baucom, Sara B. Algoe

**Affiliations:** 1 National Tsing Hua University, Hsinchu City, Taiwan; 2 The Ohio State University, Columbus, OH USA; 3 Department of Psychology and Neuroscience, University of North Carolina at Chapel Hill, CB #3270, 235 E. Cameron Avenue, Chapel Hill, NC 27599-3270 USA

**Keywords:** Human behaviour, Behavioural genetics

## Abstract

Close social connections drive mental and physical health and promote longevity. Positive, other-focused behavior like expressing gratitude may be a key mechanism for increasing close bonds. Existing evidence consistent with this claim is predominantly correlational, likely driven by challenges in causally influencing and sustaining behavior change in the context of ongoing relationships. This 5-week field experiment with daily data from couples provides the first evidence for a brief, low-cost behavioral technique to increase everyday expressed gratitude to a romantic partner. Random assignment to the gratitude expression treatment (GET) increased the amount of time couples spent co-present in everyday life, from the weeks before GET to the weeks after, relative to the control condition. This effect was mediated by the change in expressed gratitude. Voluntary co-presence is an important behavioral indicator of close bonds in non-human animals. Further analyses with a functional genotype related to the oxytocin system (rs6449182) suggest a neurochemical pathway involved in the effects of expressing gratitude. Together, this evidence bridges animal and human research on bonding behavior and sets up future experiments on biopsychosocial mechanisms linking close bonds to health.

## Introduction

Close social connections buffer against the onset and progression of psychiatric disorders, contribute to physical health, and forecast longer life^1–4^. Thus, a critical question for basic and translational research is: what are the behavioral and biological mechanisms that promote social connections? Although extensive correlational evidence documents important factors to consider, isolating causal mechanisms has proven challenging.

One key challenge is that most evidence from randomized trials focuses on couples with clinical levels of distress and employs multi-faceted behavioral interventions wherein both couple-members meet regularly with a provider to learn a variety of interpersonal skills over many weeks^5^. This makes it difficult to understand which specific behaviors may influence results. However, a recent and growing body of basic research identifies one domain of relationship behaviors that may be especially likely to actively *promote* relationships—that is, to improve their quality or closeness, as is our current goal—which is a distinct function from behaviors that repair relationships (e.g., arguing effectively^6^, forgiving^7^) or help to ensure that a partner is not in harm’s way (providing social support^8^). Behaviors in the relationship-promotion domain are positive in valence and focused on the goodness or well-being of the other person, for the person’s own sake^9,10^.

One such behavior that has garnered substantial correlational evidence in everyday life is expressed gratitude. Expressing gratitude is a characteristic behavioral response to the positively-valenced experience of *feeling* grateful for another’s kind gesture^11–13^. Methodologically sophisticated longitudinal dyadic studies show that expressing gratitude is associated with beneficial relational outcomes for both members of an ongoing relationship independently of social support and other behaviors^14,15^. Critical for the present work, gratitude is frequently expressed between romantic partners in everyday life^15–17^, and both one-time in-lab experiments as well as correlational evidence suggest that gratitude expression appears to draw the other partner in to the relationship^12,18–21^, creating a positive feedback loop between both people^22,23^. Expressed gratitude is thus a prime behavioral target in order to establish the *causal* influence of relationship behaviors on couples’ outcomes. The current investigation aims to provide evidence for just such causality: due to the positive feedback loop, we focus on having just one member of the couple express gratitude, further isolating the causal effects.

A second key challenge in causally promoting social connection in the context of real relationships is that the theoretical behavioral mechanism must be sustained—incorporated into everyday life—rather than a one-time event. However, intentional efforts to change behavior in everyday life often are ineffective^24^, especially when habits likely have formed over the course of a long-term relationship^25^. As such, our behavioral technique to promote gratitude expression in daily life was based on implementation intentions theory^26^.

Implementation intentions are plans that have the format, *If* (opportunity)—*Then I will* (response!). Meta-analysis indicates that if–then planning is a highly effective technique for promoting behavior change in a variety of domains (*d* = 0.65 in 94 tests^26^). If–then planning promotes behavior change by simulating the automatic cue-response associations that define habits and switching action control from top-down, goal-directed responding to bottom-up, stimulus control of behavior^27^. To our knowledge, implementation intentions theory has not heretofore been used to promote behavior change in romantic couples.

We focused on time spent in the physical presence of a partner as the outcome for several reasons. First, the broader animal literature uses physical proximity to a partner, often operationalized as voluntary time spent together^28^, as a behavioral index of a social bond^29^; yet it is rarely investigated in the human literature. There is ample reason to believe that grateful people may want to spend more time with the partner: the *find-remind-and-bind* theory of gratitude^22,30^ and subsequent work across labs suggests grateful people are motivated to show their beneficent partners that they, too, care about or are invested in the relationship. Spending time with the partner is an investment into the relationship^31^. Second, in both the animal and human literature, physical proximity can beneficially alter physiology^32–34^, which has cumulative implications for both emotion control and physical health. Third, time spent in physical proximity to a partner has ecological validity: in everyday life, people choose with whom and for how long they spend their time.

Fourth, time spent together links this investigation to animal research focused on the neurochemical bases of social behavior. Groundbreaking oxytocin research in prairie voles quantified how much time a female vole of a “monogamous” species spent in the physical presence of a partner with whom she had previously had a chance to bond. Importantly, she could have spent time in the presence of a different (novel) male vole^28^. The conclusion from these studies was not that voles were more *social* after receiving a central oxytocin infusion, but rather that they developed a *preference*: they chose to spend more time side-by-side with the male vole they had previously bonded with (i.e., mated/copulated)^28^. Although the human literature seeking to build upon these intriguing findings from the vole model has not yet arrived upon a consensus about whether, when, and how the oxytocin system influences relationship behaviors or outcomes, there has been great interest^35–38^. Here, we aim to build conceptually on the original vole work: Given that expressing gratitude has been characterized as bond-promoting, differential functioning within the oxytocin system might impact the degree to which an intervention to express gratitude impacts time spent with a partner.

Thus, to consider potential neurobiological pathways involved in relationship promotion, we build on our previous findings, which focused on a gene that is necessary for oxytocin secretion and normal social behavior^39^, *CD38*, and in particular, a variant (rs6449182) in this gene associated with *CD38* expression levels^40–42^. There, rs6449182 was associated with key bonding behaviors and emotions, especially the behavioral expression of gratitude (e.g., the likelihood of expressing it to a romantic partner in everyday life, via 14 nightly reports) and the reported emotion of love after a laboratory-based gratitude conversation with the partner^43^. In another study, urinary oxytocin was associated with ratings of love after gratitude conversations^44^, suggesting that these genetic associations may indeed be a result of differences in oxytocin signaling. For further discussion of other variants in the CD38 gene and social relationships, see the Supplementary Information.

**Figure 1:**
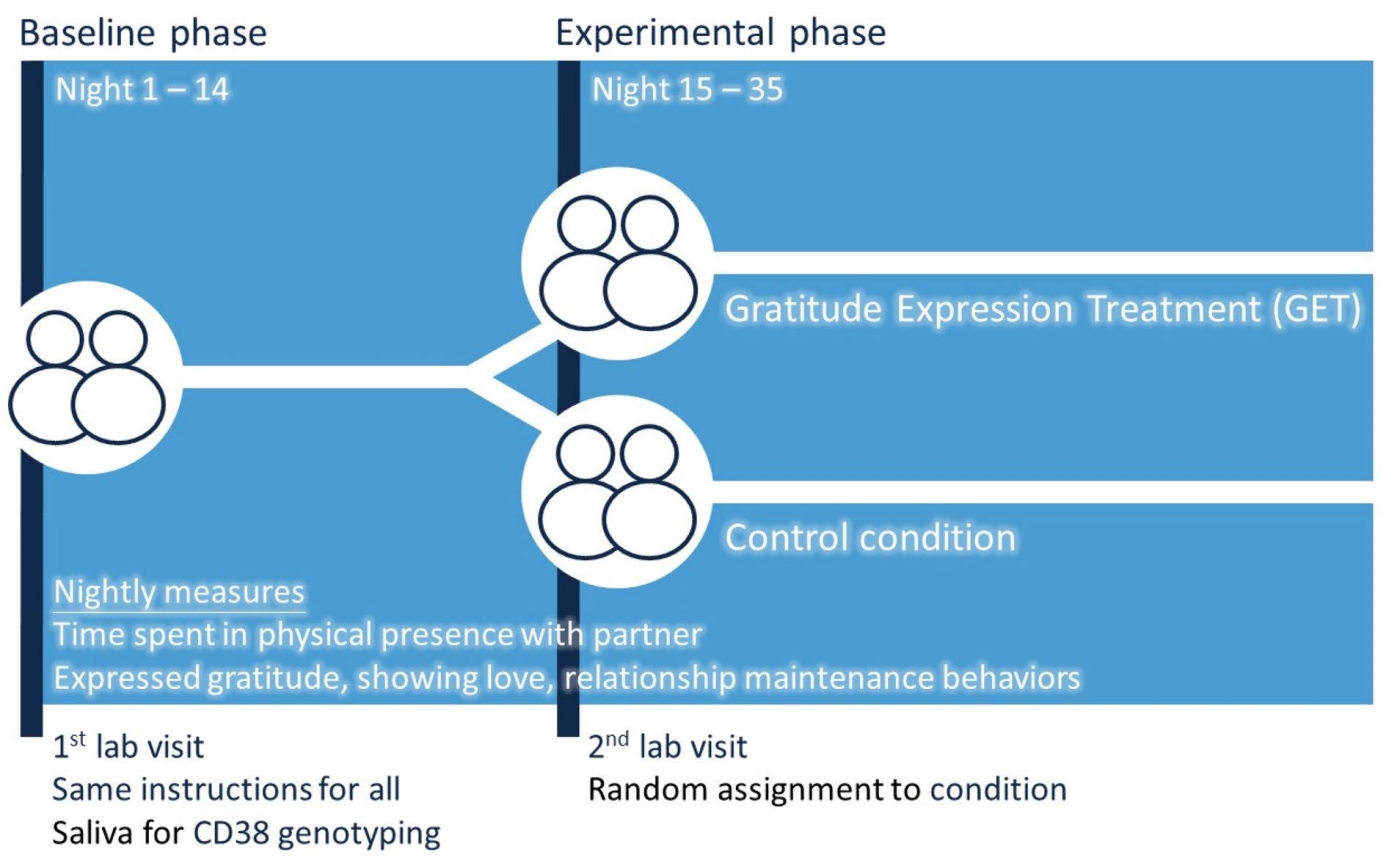
Overview of 5-week experimental protocol. 250 individuals from 125 couples completed the 5-week protocol, resulting in 28.65 of 35 possible nightly reports per person, on average, or 7162 observations.

**Figure 2:**
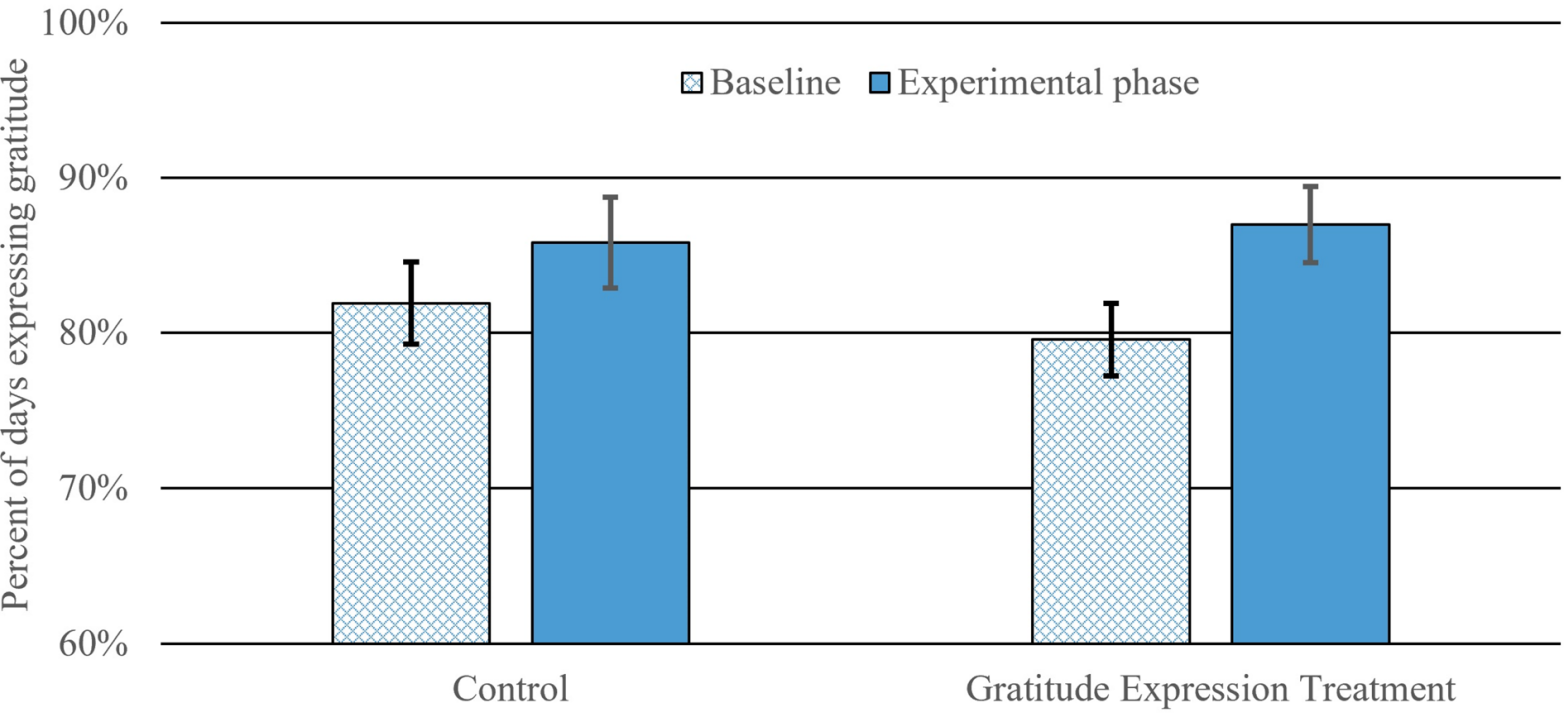
Phase × condition interaction predicting expresser’s nightly report of expressing gratitude. For ease of interpretation, raw values (% of days within the baseline vs. experimental phase) and their SDs are depicted rather than model estimates.

**Figure 3:**
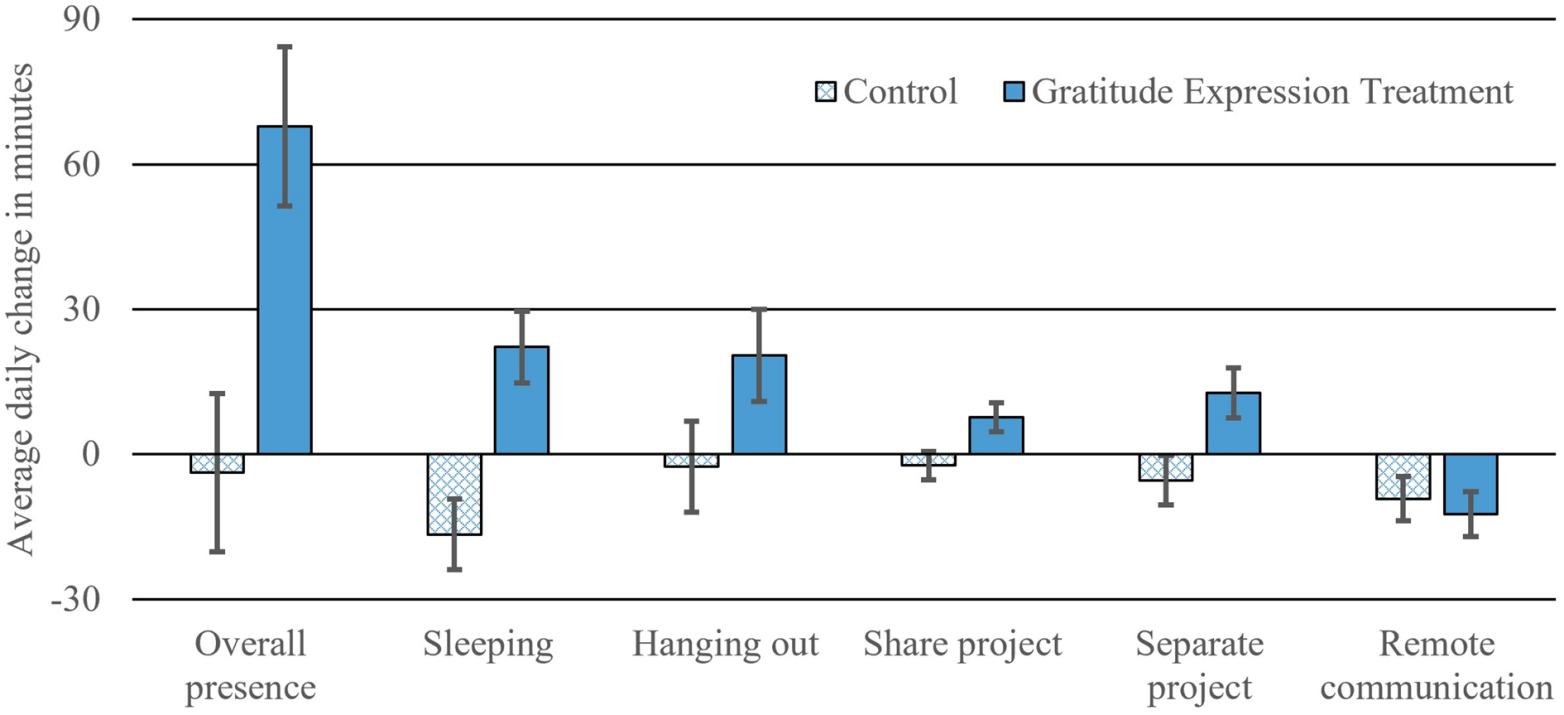
Change in time spent from the baseline to the experimental phase of the study (and the SE of the change), depending on experimental condition, for each way the couple may have been physically co-present.

We present data from a 5-week field experiment with romantic couples whose members independently reported on their behavior and time spent in the physical presence of the partner, nightly. See Fig. 1 for procedural overview. After 2 weeks of reporting (baseline phase), one member of half the couples was randomized to briefly make a plan to express gratitude to the partner more frequently (gratitude expression treatment; GET), using a private 5-min task. We test the effects of GET on subsequent change in daily expressions of gratitude (Fig. 2) and on daily time spent with the partner (Fig. 3) in the post-treatment weeks (experimental phase) compared to changes in the control condition (i.e., phase × condition interaction); we also assess whether expressed gratitude mediates the latter effect.

Further, we examine whether people with the rs6449182 genotype associated with those who are already likely to express gratitude more frequently in daily life in prior work^43^ benefit most (a potential sensitivity effect^45^) or alternatively, least (a potential ceiling effect^46^), from GET. Testing this gene × experimental-effect interaction (statistically, a gene × phase × condition interaction) is relevant to understanding basic research questions about the role of the oxytocin system in human pro-relationship behavior by carefully manipulating a potential behavioral mechanism through which the oxytocin system may influence change in time spent co-present. This statistical interaction test is also relevant to a fundamental question in translational research: *for whom* do behavioral interventions work^47,48^?

## Results

### Data analysis overview

Four sets of multi-level models that build upon one another were used to analyze the data. Modeling details are described in the Methods; in order of model complexity, models focus on: estimating descriptive information about outcomes, direct and conceptual replication of main effects of rs6449182 on nightly outcomes during the baseline phase, testing the effect of condition on change in time spent with the partner, and the rs6449182 × condition × phase interaction. All significance tests were two-sided.

### Manipulation check: GET increases grateful behaviour

The phase by condition interaction significantly predicted the expresser’s likelihood of reporting they thanked the partner on a given night (est. = 0.14, *SE* = 0.05, *df* = 3572, *t* = 2.82, *p* = 0.005, *OR* = 1.15, [CI 95%] = [1.04, 1.27], demonstrating that GET increased thanking behavior relative to baseline (main effect of condition, est. = 0.03, *SE* = 0.14, *df* = 3572, *t* = 0.23, *p* = 0.815, *OR* = 1.03, [CI 95%] = [0.78, 1.37]; main effect of phase, est. = 0.24, *SE* = 0.05, *df* = 3572, *t* = 4.81, *p* < 0.001, *OR* = 1.27, [CI 95%] = [1.15, 1.40]). Further, tests of the simple effects within condition are illustrated in Fig. 2, showing that expressers in the control condition did not change their likelihood of expressing gratitude from the baseline phase to the experimental phase (i.e., 81.9% to 85.8%; est. = 0.09, *SE* = 0.07, *df* = 1829, *t* = 1.37, *p* = 0.170, *OR* = 1.10, [CI 95%] = [0.96, 1.25]). By contrast, expressers in GET significantly increased expressions of gratitude from 79.6 to 87.0% (est. = 0.38, *SE* = 0.07, *df* = 1743, *t* = 5.09, *p* < 0.001, *OR* = 1.46, [CI 95%] = [1.26, 1.69]).

### Effect of GET on everyday time spent with partner

Descriptive information about daily time spent in the physical presence of the partner can be found in Table S1. Compared to the control condition, couples in GET reported spending significantly more time in the physical presence of one another in the 3 weeks after one member made a plan to express gratitude more frequently compared to the 2 weeks prior to the manipulation (see Table 1; Table S3 contains the main effects of Phase and Condition that are omitted in Table 1). The overall two-way interaction was statistically significant (the first bolded row in Table 1), as was the estimated increase in time spent from baseline to experimental phase just within GET (the rows under the row for the two-way interaction); results imply an estimated 68 min average daily increase in time spent together in the experimental condition, compared to control (see Fig. 3). Supplementary Information provides evidence that it does not matter which partner reports time spent, so the finding was not due to reporting bias from the partner who received the manipulation, nor was the effect moderated by expresser sex.

**Table 1:** Experimental manipulation effects and time changes in each condition.

	Est	SE	df	t	*p*	[CI 95%]	
Overall time in proximity*	17.94	5.82	1948.40	3.08	.002	6.53	29.34
Control	− 3.87	16.37	1940.82	− 0.24	.813	− 35.97	28.23
GET*	67.87	16.53	1955.87	4.11	< .001	35.44	100.30
Sleeping*	9.68	2.61	2072.60	3.71	< .001	4.56	14.79
Control*	− 16.53	7.34	2048.07	− 2.25	.024	− 30.92	− 2.15
GET*	22.18	7.42	2096.96	2.99	.003	7.63	36.72
**Hanging out**^†^	5.76	3.36	2046.18	1.71	.087	− 0.83	12.35
Control	− 2.59	9.45	2025.19	− 0.27	.784	− 21.13	15.94
GET*	20.43	9.56	2066.96	2.14	.033	1.68	39.18
Share project*	2.50	1.05	2274.24	2.38	.018	0.44	4.57
Control	− 2.39	2.95	2221.30	− 0.81	.417	− 8.18	3.39
GET*	7.62	3.01	2326.50	2.53	.011	1.71	13.53
Separate project*	4.52	1.82	2004.88	2.49	.013	0.96	8.08
Control	− 5.40	5.10	1967.38	− 1.06	.289	− 15.40	4.60
GET*	12.67	5.17	2042.16	2.45	.014	2.52	22.82
**Remote communication**	− 0.79	1.62	1771.96	− 0.49	.626	− 3.98	2.39
Control*	− 9.25	4.56	1749.82	− 2.03	.043	− 18.20	− 0.30
GET*	− 12.42	4.62	1793.29	− 2.69	.007	− 21.48	− 3.36

*^,†^*p* < .05 and .10 respectively; each type of time estimate from multi-level models shows the manipulation effect on the type, based on the two-way interaction between experimental manipulation and phase; non-bolded rows for control and GET conditions are the model-estimated changes in time spent in that type of time, within each condition (i.e., the simple effects), and scaled to the unit of minutes.

Additionally, this pattern held for each exploratory measure of daily time spent together (Table 1 and Fig. 3): sleeping next to one another, hanging out, working on a shared project, or working on separate projects in the same space. GET did not change the amount of time spent “with” the partner while physically absent: both conditions significantly decreased on this variable and the difference in the decrease (i.e., the interaction term) was not significant.

Finally, we tested the theorized behavioral mechanism of expresser’s gratitude, using a model appropriate for the binary mediator (express or not) and continuous outcome variable^49^. Supporting our prediction, the indirect effect of the manipulation on time spent with the partner through expressers’ gratitude expressions was significant, Aroian *Z* = 2.78, *p* = 0.006 (direct effect, est. = 15.70, *SE* = 5.70, *df* = 1892.20, *t* = 2.75, *p* = 0.006, [CI 95%] = [4.52, 26.88]).

### rs6449182: direct and conceptual replication of Algoe and Way (2014)

Table 2 shows rs6449182 was significantly associated with likelihood of expressing gratitude and showing love as well as relationship satisfaction in the 2-week baseline phase, directly and conceptually replicating prior research^43,44^. This provides additional confidence of the relevance of this SNP to the current research question about the role of oxytocin signaling in bonding behavior. In exploratory analyses of conceptually different variables, rs6449182 was not significantly associated with the suite of daily relationship maintenance behaviors or overall time spent with the partner. These effects were not moderated by participant sex (see Supplementary Information).

**Table 2:** Associations between rs6449182 and baseline daily relationship measures.

	Est	SE	df	t	*p*	OR	[CI 95%]	
Relationship satisfaction*	− 0.18	0.08	186.76	− 2.16	.032		− 0.34	− 0.02
Gratitude*	− 0.38	0.16	3091.00	− 2.41	.016	0.68	0.50	0.93
Love*	− 0.58	0.23	3091.00	− 2.52	.012	0.56	0.36	0.88
Relationship maintenance	− 0.01	0.17	230.58	− 0.71	.476		− 0.05	0.02
Time spent (min)	8.70	18.17	713.32	0.48	.632		− 26.97	44.37

*p < .05; Est. represents an unstandardized regression coefficient from a multi-level model regressing the dependent measure from the 14-night baseline phase on genotype.

### GET moderates association between expresser rs6449182 genotype and time spent with partner

We focused only on expresser (not partner) genotype because expressers were the individuals randomly assigned to GET. Analyses reveal a significant three-way interaction between expresser genotype, condition, and experimental phase on both partners’ reports of overall time spent (see Table 3 and Table S6).

**Table 3:** GET effects on overall time spent within different expresser rs6449182 genotypes.

	Est	SE	df	t	*p*	[CI 95%]	
Condition × Phase*	17.45	5.85	1920.70	2.98	.003	5.98	28.92
× rs6449182*	− 12.93	5.80	1938.34	− 2.23	.026	− 24.30	− 1.55
CC*	104.94	28.35	1918.61	3.70	< .001	49.35	160.55
CG	17.79	32.86	1937.92	0.54	.588	− 46.65	82.22
GG	− 69.38	66.41	1941.77	− 1.05	.296	− 199.62	60.86

*p < .05. The first row of condition × phase interaction shows the experimental effect; the second row shows how GET moderates the association between rs6449182 and time spent; the following three rows present the results of the simple effects of the condition × phase interaction within each expresser genotype.

Decomposition of this three-way interaction (see Table 3) further into a two-way interaction within each genotype indicates that the manipulation only significantly affected couples in which the expresser had the CC genotype, but not expressers with CG or GG. Expressers with the CC genotype comprised about 2/3 of the sample and were most likely to express gratitude during the baseline phase (Table 2; see also Algoe and Way^43^). Breaking down that significant two-way interaction for CC individuals, the simple effects within condition show that couples in GET significantly increased the overall time spent together (est. = 83.31, *SE* = 20.30, *df* = 1914.23, *t* = 4.11, *p* < 0.001, [CI 95%] = [43.51, 123.12]), whereas those in the control condition did not (est. =  − 21.63, *SE* = 19.79, *df* = 1923.15, *t* =  − 1.09, *p* = 0.275, [CI 95%] = [  − 60.46, 17.19]). The Supplementary Information presents results of supplemental exploratory analyses showing these effects were not further moderated by expresser ethnicity or sex.

## Discussion

This 5-week field experiment involving both members of ongoing romantic relationships documented causal increases in a novel outcome—daily time spent in the physical presence of a partner—that has implications for health. It did so by merging two theories. First, drawing from the *find-remind-and-bind* theory of gratitude^22,23,30^, it focused on one everyday behavior, expressed gratitude, that is relationship-promoting in correlational work^15^ and that we predicted would influence *both* members of the couple if implemented by just one couple-member in a randomized experiment. Second, drawing from implementation intentions theory^26^, it helped people create new habits by making a plan^27^ to express gratitude frequently in response to specified cues. Indeed, the indirect effects suggest that expressers in the experimental condition, compared to control, drove increases in both couple-members’ reports of time spent with the partner by increasing expressers’ frequency of expressing gratitude to the partner in daily life. By focusing on one behavior implemented by one person in the couple, this novel approach for the close relationships literature provides a compelling method for identifying behavioral mechanisms for promotion of interpersonal connection in the context of ongoing relationships.

Specifically, rather than focusing on repairing damaged relationships or helping a partner during a challenging time, we targeted a theoretically-specified, relationship-promoting behavior that occurs in a wide variety of relationships, expressing gratitude^10^. Moreover, while a few promising experiments have recently tested interventions for couple outcomes that do not require repeated contact with a clinician to work through issues or learn a series of skills^50,51^, those studies involve both members of the couple actively trying to change their relationship behavior. In contrast, the brief instructions in GET were administered to just one member of the pair, substantially increasing scientific inference regarding who and what behavior was driving the effect.

This causal evidence for the effectiveness of promoting gratitude expressions in daily life opens the door to future research in non-clinical populations. Given the centrality of good relationships for functioning and survival, this type of basic mechanistic research will be essential going forward. As one example, a key strength of the experimental design is that we manipulated behavior in just one person, yet the downstream consequences were relevant to both. In practice, a growing array of evidence suggests that both the expresser and the person receiving the expression can be favorably impacted by a gratitude expression: both people are drawn to the relationship^20,52,53^. Future research would be warranted to disentangle whether, once gratitude is expressed, one or the other or both partners are initiating the time spent together. Further, the topic of *how* good relationships contribute to long-term health outcomes traditionally focuses on chronic stress pathways^54–56^ rather than salubrious pathways. Yet salubrious pathways may better characterize high-quality relationships^10^. Thus, the present evidence for the value of if–then planning to cause behavioral change over time through random assignment to express gratitude offers a useful, theoretically-grounded approach for future work in this important area of research.

Regarding the novel methods, it is noteworthy that a 5-min online task led to significant behavior change. This is important preliminary validation of implementation intentions in a novel context where behavior change is notoriously difficult and provides mechanistic insight into how and why good relationships might contribute to mental and physical health. It may be valuable in future trials to further corroborate the behavioral change of expressing gratitude through non-self-report means, as well as to dismantle the three components of GET (i.e., setting a gratitude expression goal, forming an if–then plan, and rehearsing the plan) to determine the key ingredients of the instructions. Additionally, the novel outcome that ties the human to the animal literature—voluntary time spent in the physical presence of the romantic partner—can be readily assessed in ecologically valid ways, verified with partner reports, and in future work, linked with mental and physical health outcomes. For example, more time together with a loved partner could beneficially influence physiological co-regulation; could provide opportunities for physical affection, feeling loved, or accomplishing shared goals; or simply increase the likelihood that someone will notice when a partner has fallen in the shower. Those additional 60-plus minutes together per day for couples in the experimental condition provide a wealth of opportunities.

Additionally, the present findings build an important bridge from the animal oxytocin^28^ literature to humans, simultaneously addressing basic research questions about the neurochemical bases of social behavior and providing evidence about *for whom* the bonding manipulation might be most effective. The direct replication of the association between rs6449182 and expressed gratitude as well as this SNP’s association with daily expressions of love, which conceptually replicates a prior association of this SNP with feelings of love after a gratitude conversation, lend confidence to the relevance of this SNP to the focal experimental manipulation of expressed gratitude. Further, using repeated daily measurements completed in close temporal proximity to events—indeed, more than 7000 observations—offers greater power and reliability than the self-report inventories typically used in contemporary genetic association studies that have larger sample sizes^57^. However, we view these findings as an initial step that needs supplementation with experimental manipulation of brain oxytocin signaling. Such an approach could confirm the proposed pathways derived from the present data: analogous to prior vole findings, we theorize that oxytocin signaling in the brain, for which *CD38* is a key regulator, is modulating the response to the bonding opportunity we provided in GET. Proximally, the pattern of data suggests that making the plan helped people already inclined to express gratitude and be other-focused (CC genotype) close the gap between their intentions and behaviors. This is in line with previous research indicating that planning mainly benefits people who already favor performing the behavior^58^. Once implemented, there are likely perceptual mechanisms at play to foster closeness with this particular partner^44,59,60^, leading them to forego other things they could be doing with their time, either alone or with other people. (See Supplementary Information for similar findings based on rs3796863, another variant in *CD38*.) Importantly, although the key finding of condition on change in time spent was modulated by *CD38* status, the effective genotype represented the majority of expressers in our sample (63.6%); that is, consistent with the broad literature on expressed gratitude^15,17^, most people have this capacity.

In conclusion, building on insights from close relationships research and implementation intentions theory, this low-cost behavioral technique of making an if–then plan to express gratitude shows great promise for both basic and translational research in a domain that is central to human mental and physical health: social relationships. Moreover, the novel, yet well-grounded links with non-human animal literature—namely, focusing on the value of physical proximity in a free-range scenario and the role of the oxytocin system in influencing bond-promoting behaviors—provides a solid foundation for future work in this area.

## Method

### Participants

Couples were recruited for a 5-week study on “Everyday Couple Interactions”; 136 couples (*N* = 272) attended the first laboratory session after individually screening and consenting to participate. Informed consent was obtained from all participants in this human subjects protocol, which was approved by the Institutional Review Board at UNC Chapel Hill (#13-1976) and carried out as approved under the Board’s guidelines and regulations. See Supplementary Information for full demographic information and inclusion criteria, as well as the test for the manipulation-moderated rs6449182 effects by different ethnicities in Table S7.

### Experiment overview (Fig. 1)

Couples attended three laboratory sessions together: at baseline, 2 weeks later when the experimental manipulation was administered, and again 3 weeks later. Each person independently completed online nightly questionnaires for the full 5 weeks. At the second laboratory visit, couples participated in a videorecorded gratitude expression protocol^18^ that set the stage for the manipulation by (a) identifying one randomly-selected member of the couple as the gratitude expresser (and the other as the target of that expression), and (b) making it seem natural to the expresser that we would ask them to focus on expressing gratitude in the subsequent weeks. Specifically, at the end of the session, couple members were in separate rooms to complete final measures; while there, the couple-member who had expressed gratitude in the lab conversation was randomly assigned to receive additional instructions (gratitude expression treatment; GET condition) or not (control condition). In GET, the expresser created an if–then plan to express gratitude to the partner more frequently in the subsequent weeks.

### Procedural compliance

Nightly data from the 2-week *baseline* phase were used to test direct and conceptual replication of prior work^35^. This phase produced 3332 nightly reports from 269 participants (88.50% compliance) for analyses involving only these data. The subsequent 3 weeks of nightly reports represent the *experimental* phase in this within-subjects aspect of the experimental design (phase: baseline vs. experimental). The full 5-week within-between experimental design will be used to test for within-subjects *change* in time spent together from baseline to the experimental phase, as a result of the manipulation. The latter test only included participants who completed the experimental protocol, which reduced the sample by 10 couples (8 did not attend the second session and receive the manipulation, 2 did not attend the final session); of these, 63 couples were randomly assigned to the experimental condition and 62 to the control condition. Therefore, these analyses and conclusions rely on the 7162 reports from 250 participants who completed the full 5-week protocol; of these, 3134 (89.57% compliance) and 4028 (76.72% compliance) nightly reports are from the baseline and experimental phases, respectively.

### Gratitude expression treatment (GET)

At the end of the second laboratory session, while expresser and target were separated to complete final questionnaires, the GET manipulation was administered via Qualtrics online survey software. Expressers who were randomly assigned to GET received the following prompts, whereas those in the control condition did not. Making the plan to express gratitude was presented in four screens through which the participant advanced at their own pace. Verbatim text is below.

**Screen 1:**

Research in our lab shows that people who express their gratitude to the partner when they appreciate things the partner does tend to be happier in their relationships. **We’d like you to consider doing this for yourself in the next few weeks to see how it goes.**

There is no need to sit down and have a long conversation with your partner like you just had here in the lab. However, no matter how often you typically do say thanks when your partner does something you appreciate, we’d like you to consider doing this even more often than you do now.

Specifically, during the next three weeks, when you ***feel*** grateful or appreciate something your partner has done for you, take the opportunity to ***express*** your gratitude.

First in the space below, consider some of the things your partner has done in the last week that you appreciated. Briefly list as many here as you wish—your partner’s actions can be big or small, and remember your partner will never see this list, it is for your reference:

[Open-text box here].

**Screen 2:**

Second, it helps to have a plan. Please tell yourself the following:

**“If my partner does something that I appreciate, then I will express my gratitude.”**

Please repeat the plan to yourself several times—until you can say it fluently from start to finish, without reading it.

**Screen 3:**

In the spaces below, please type your plan IN CAPS,

If [open-text box here].

Then [open-text box here].

**Screen 4:**

**One final note:** We are giving you this extra encouragement because you were the person randomly assigned to express your gratitude in the face-to-face conversation you just had with your partner. Your partner is not receiving these (or any) instructions, and your partner doesn’t know we are giving you this extra encouragement to avoid putting pressure on you. **We simply want you to do this as a personal project and see how it goes to pay more attention to—and find more opportunities to—thank your partner for a few weeks.** At the end of the three weeks, you may enjoy chatting with your partner about it, but for now we encourage you to try it on your own so you can see what works best for you.

### Nightly measures

Each night each participant independently estimated the amount of time they spent in the physical presence of their partner in the prior 24 h (“i.e., you were in the same room with the person, whether awake or sleeping”). This was the focal dependent measure, “overall time spent”. For exploratory purposes, they estimated time spent together in five additional subcategories: sleeping, hanging out, working on a project together, doing their own thing (in the same space), and time physically apart but communicating.

Relationship behaviors were assessed with a checklist (yes/no; see Supplementary Information for items). One behaviour—expressed gratitude to the partner—was included as a manipulation check for expressers and potential mediator of our predicted effects on time spent with the partner. Furthermore, responses to this item during the baseline phase will be used to test for direct replication of prior research on rs6449182^43^. A second item—“I showed my love for him/her”—will be used to test for conceptual replication of prior research on rs6449182 and urinary oxytocin^43,44^. The other 14 relationship behaviors were averaged daily as a “relationship maintenance” composite to explore generalizability of prior rs6449182 effects to theoretically broader outcomes. Likewise, participants reported their daily satisfaction with the relationship, which will be used to test for conceptual replication of prior research on rs6449182 and urinary oxytocin^43,44^.

### Genotyping

Salivary DNA collection and extraction as well as the genotyping platform were the same as in our prior research^43^. Similarly, rs6449182 was coded in an additive manner (CC = 0, *n* = 149; CG = 1, *n* = 79; GG = 2, *n* = 17) to match the preponderance of data on the effects of this SNP on *CD38* expression. Genotyping results conformed to Hardy–Weinberg equilibrium (*χ*^2^ = 2.36, *df* = 1, *p* = 0.124), and the genotyping call rate was 99.3%. Please see Supplementary Information, specifically, Tables S9 to S12 for more information about exploratory analyses regarding a second *CD38* SNP, rs3796863, which was not robustly correlated with bonding-relevant measures in our prior research^43^ but has been of recent interest in the literature^61,62^. These were the only 2 SNPs that were measured.

### Modeling details

We describe the four data analytic models here in order of model complexity, but present results in order of the research questions. First, following the suggestion of modeling dyadic daily diaries using two-level cross-lagged models in which daily diaries are nested in dyads^63^, we used a two-level cross-lagged *null* model (i.e., without predictor and with a dyad-level random intercept, a random slope of participants’ roles, and auto-correlated day-level residuals) to estimate the basic descriptives of each variable of shared time (so ${DV}_{ij}={\beta }_{0}+{\varepsilon }_{0j}+{\varepsilon }_{1j}{role}_{ij}+{\varepsilon }_{ij}$; the model was changed to a logistic model below for binary DVs, e.g., daily gratitude expression). This model was only conducted on the subset of data that it was intended to describe, because it was for descriptive purposes. For instance, when estimating the descriptives of variables during the pre-manipulation *baseline phase*, the model was fit only to the pre-manipulation data for those variables, as opposed to, say, being fit to all data and generating simple estimates for the focal phase. Finally, although role (expresser versus target of the expression) was used as a variable to fit the model, the covariance between random effects (i.e., the role random slope and the dyad-level random intercept) was set to 0 so that role only served to group same-individual data together (to make the model *cross*-lagged), because we had no interest in exploring the difference between expressers and targets.

Second, to test for direct and conceptual replication of prior main effects for relationship satisfaction, expressed gratitude, and love^43,44^, we added rs6449182 (called “snp” in the model) to the above descriptives model as a predictor (so ${DV}_{ij}={\beta }_{0}+{\beta }_{1}{snp}_{j}+{\varepsilon }_{0j}+{\varepsilon }_{1j}{role}_{ij}+{\varepsilon }_{ij}$). This model was only fitted to baseline reports. For exploratory purposes, we also tested associations between rs6449182 and the relationship maintenance composite score as well as overall shared time on a given day.

Third, we built a model to assess the effects of the manipulation on how participants spent their time: we added two dummy codes, one for experimental condition (GET = 1, control = − 1) and the other for phase (baseline =  − 1, experimental = 1), as well as their interaction in the descriptive model (so ${DV}_{ij}={\beta }_{0}+{\beta }_{1}{phase}_{ij}+{\beta }_{2}{cond}_{j}+{\beta }_{3}{(phase\times cond)}_{ij}+{\varepsilon }_{0j}+{\varepsilon }_{1j}{role}_{ij}+{\varepsilon }_{ij}$). The interaction between condition and phase was hence the critical test of the effects of the manipulation and was used on the focal dependent measure of overall time spent with the partner; we also explored each way the couples might have shared time. As a manipulation check, we also fit this model to expressers’ reported gratitude expressions. Finally, we used this model to examine whether the anticipated changes in expressed gratitude mediated the effects of the manipulation on change in overall shared time, following the suggested mediation test of associations^49^ where the DV is continuous but the mediator is binary to assess whether the indirect effect of the manipulation on total daily shared time between partners (continuous) via expressers’ expressing gratitude (binary) was larger than 0. It might be worth noting that, even though both partners in a couple reported the time they shared with one another on a given day independently and, thus, their reports might not match perfectly, the reports did highly correlate as expected, as observed by the fact that their independence (estimated by the variance of the role random slope) was shown as statistically superfluous in many of the shared-time models. Specifically, the variance of this role random slope was shown redundant and inestimable for overall shared time, sleeping together, and hanging out together in the descriptives model, showing that the two roles—the partners—in a couple were almost equivalent in the shared time they reported for these aspects on a given day. By contrast, the variance was estimable for working on shared projects, separate projects, and remote communication. This might be because the baseline amounts of these three domains of shared time—as shown in Table S1—are relatively little. Relatively, then, any report inaccuracy and difference between partners would seem large and meaningful for those three variables: rounding 1.5 h to 2 h means a larger error than rounding 10.5 h to 11 h. Nonetheless, the important thing here is that our analysis took the variability into account when needed by including the random slope in the model as opposed to dropping it (see its raw statistics in Table S1), and this model setup should not affect the estimation of fixed effects (e.g., potentially inflating statistical power; this was not the case because, as can been seen in Table S2, the *df*s of the tests were adjusted, as in all multi-level models, here reduced because of the interdependence). Further, given there was little to no between-partner discrepancy (see Table S2), the effects on shared time reported in text replicated when we fit the model only to expressers or to targets.

Fourth, to explore the potential moderating effects of GET on the association between the expresser’s rs6449182 genotype and change in time spent with the partner, we added the expresser’s rs6449182 genotype (standardized) and its interaction with the two dummy codes in the above manipulation-effect model (so ${DV}_{ij}={\beta }_{0}+{\beta }_{1}{phase}_{ij}+{\beta }_{2}{cond}_{j}+{\beta }_{3}{(phase\times cond)}_{ij}+{\beta }_{4}{snp}_{j}+{\beta }_{5}{(snp\times phase)}_{ij}+{\beta }_{6}{(snp\times cond)}_{j}+{\beta }_{7}{(snp\times phase\times cond)}_{ij}+{\varepsilon }_{0j}+{\varepsilon }_{1j}{role}_{ij}+{\varepsilon }_{ij}$). We focused on the three-way interaction because it measured the effect of the tested SNP on the manipulation-effect phaseXcondition interaction. The same model was used to explore the potential moderating effect of expresser’s rs3796863 genotype (see Supplementary Information).

Regarding effect sizes, although there have been significant advances in how to estimate and interpret effect sizes from multilevel models in recent years^64^, this is still a developing and unsettled issue, in part because there are multiple effect sizes associated with each explanatory variable in the model. It is therefore suggested that researchers find the most informative indices of effect sizes for their research contexts^64^. As such, because the measure was time in actual minutes (e.g., as opposed to an arbitrary unit such as a Likert-type scale), we calculated the model-implied minutes increased from before to after the manipulation with their estimated CIs as the index of effect sizes. We believe these are both natural and readily interpretable for readers.

## Supplementary Information

Supplementary Information.

## Data Availability

These data are not currently available for public use as they contain information that could potentially compromise research participant privacy. Please direct any communications concerning data availability to the corresponding author.

## Code availability

Analysis syntax is available for public access at 10.15139/S3/9PYDTN.
