# Supplementary material for: Implementation intentions to express gratitude increase daily time co-present with an intimate partner, and moderate effects of variation in CD38
Source: Sci Rep. 2022 Jul 9;12:11697. doi: 10.1038/s41598-022-15650-4 (PMC9271060; doi:10.1038/s41598-022-15650-4)
Supplement: Supplementary file 1 — Supplementary Information. [file 41598_2022_15650_MOESM1_ESM.docx]

**TABLE OF CONTENTS**

[METHOD 2](#_Toc103720946)

[Participants 2](#_Toc103720947)

[Nightly measures 2](#_Toc103720948)

[Specific items from the nightly questionnaire 3](#_Toc103720949)

[Cleaning of nightly data 3](#_Toc103720950)

[Experimental manipulation 4](#_Toc103720951)

[RESULTS 5](#_Toc103720952)

[Table S1. Descriptive Statistics of Daily Physical Proximity 5](#_Toc103720953)

[Table S2. Supplementary Descriptive Statistics of Daily Physical Proximity 6](#_Toc103720954)

[Table S3. Main Effect Statistics for the Models estimating GET Effects on Time Changes for Phase and Condition (supplement to manuscript Table 1 and Figure 2) 6](#_Toc103720955)

[Table S4. GET Effects for Members of Couples Where the Expressers were of Different Sexes 7](#_Toc103720956)

[Table S5. Moderation of Sex on the Associations Between rs6449182 and Baseline Daily Relationship Measures 7](#_Toc103720957)

[Table S6. GET Effects for Individuals with Different rs6449182 Genotypes 7](#_Toc103720958)

[Table S7. Testing for Manipulation Effects by Ethnicity 8](#_Toc103720959)

[Table S8. GET Effects on Relationship Satisfaction for Phase and Condition 10](#_Toc103720960)

[EXPLORATORY RESULTS FOR rs3796863 11](#_Toc103720961)

[Background 11](#_Toc103720962)

[Genotyping 11](#_Toc103720963)

[Results 11](#_Toc103720964)

[Table S9. Associations Between rs3796863 and Baseline Daily Relationship Measures 11](#_Toc103720965)

[Table S10. Manipulation Effects for Individuals with Different rs3796863 Genotypes 12](#_Toc103720966)

[Table S11. Manipulation Effects on Overall Time Spent within Different Expresser rs3796863 Genotypes 12](#_Toc103720967)

[Table S12. Testing for Manipulation Effects by Ethnicity with rs3796863 13](#_Toc103720968)

[rs3796863 and Gratitude: Comment 13](#_Toc103720969)

[References 15](#_Toc103720970)

# **METHOD**

## Participants

***Eligibility.*** Participants were required to have been involved in an exclusive romantic relationship for at least one year, be at least 18 years old, and needed to have daily access to the internet. Additionally, participants must not have been recently diagnosed with anxiety or depression, nor could they be taking steroid medication; women were pre-menopausal, not currently pregnant or nursing, not pregnant in the prior six months, and had not had an oophorectomy.

***Demographics.*** Couples were heterosexual (50% female), and had been together for an average of 4.18 years (*SD* = 5.16); 61.0% were dating exclusively, 10.3% were engaged, 26.8% were married, 1.9% reported another status. Twenty-four individuals indicated having children (among these, *M* children = 2.38; *SD* = 1.25). Finally, participants were on average 27.07 years old (*SD* = 9.85); 65.4% reported being White/Caucasian, 11.0% African-American, 4.8% East Asian, 2.6% South Asian, 0.4% American or Alaskan Native, and 15.8% were of other ethnicities; 8.5% of participants self-identified as Hispanic.

## Nightly measures

***Time spent in physical presence with partner.*** Each night, participants answered the question “In the past 24 hours (since you last completed this questionnaire), about how much time did you spend in the *physical presence* of your partner (i.e., you were in the same room with the person, whether awake or sleeping)?” This is the focal dependent measure.

Subsequently, for exploratory purposes, they were asked about four potential ways they might have spent that time together: “Of that time, how much, if any, was spent sleeping next to one another?”, “… ‘hanging out’ together (e.g., eating, watching a movie, going for a walk, etc.)?”, “… working on a project together (e.g., housework)?”, and “… doing your own thing (e.g., working on independent projects but in the same space)?” Finally, participants were also asked about “social” activities while physically absent: “How much time did you spend communicating/hanging out with your partner together through other means (e.g., phone, text message, facetime, webcam)?” This is referred to as “remote communication” in the main manuscript. Participants responded to each of these six items by indicating both the number of hours and minutes spent. These estimates were converted to total time in minutes for each measure.

***Relationship satisfaction.*** Participants reported the extent to which their relationship that day was “terrible” (1) to “terrific” (9), on a 9-point scale, with higher values representing greater relationship satisfaction that day^1^, which we use to test for conceptual replication of prior research.

***Expressed gratitude, showing love, and “relationship maintenance behaviors”.*** From within a checklist list of 16 behaviors a participant may have enacted toward the partner each day (reported as yes = 1, no = 0), we focused on two for direct and conceptual replication of prior findings on gratitude and love^2^: “I thanked my partner for something he/she did that I appreciated” (direct replication) and “I showed my love for him/her” (conceptual replication). Expressing gratitude will also be used as a check of the effectiveness of the manipulation. We note that this is an exploratory analysis because the regularity of expression may not match the time scale of this item; for example, if most people in these satisfied couples already express gratitude daily, and GET increased behavior to multiple times per day, the analysis may not capture behavior change.

Additionally, we explored a broader set of behaviors characterized as “relationship maintenance” behaviors in prior literature^3^, to probe the specificity of the previously documented effects: the additional 14 items were averaged each day to create a composite score, with high scores representing greater engagement in maintaining the relationship. Example items include “Acted cheerful and positive when with him/her” and “Helped equally with tasks that needed to be done.” The next page contains the specific items used in the study.

## Specific items from the nightly questionnaire

Please indicate whether or not each event occurred today:

I attempted to make our interactions very enjoyable.

I was cooperative in the ways I handle disagreements between us or have tried to solve a conflict.

I tired to build up his/her self-esteem, including giving him/her complements, etc.

I was very nice, courteous, and polite when we talk.

I acted cheerful and positive when with him/her.

I tried to be romantic, fun, and interesting with him/her.

I was patient and forgiving of him/her.

I encouraged him/her to disclose thoughts and feelings to me.

I simply told him/her how I felt about our relationship.

I stressed my commitment to him/her.

I implied that our relationship has a future.

I thanked my partner for something he/she did that I appreciated.

I showed my love for him/her.

I helped equally with tasks that need to be done.

I offered to do things that aren’t ‘my’ responsibility.

I did my fair share of the work we have to do.

## Cleaning of nightly data

***Inclusion of nightly report.*** All nightly reports must have met the following criteria to be included. First, they had to be “completed” online survey entries, as opposed to partially completed entries that were automatically terminated by the survey software when the data collection period was closed. Second, because the reports were meant to be end-of-day reflections on the prior 24 hours (daytime), and participants received those instructions, reports submitted between 6:30am and 4pm were removed; those submitted between midnight and 6:30am were considered reports for the prior day. Third, if there were multiple reports recorded on a given day, only the last entry of a participant on a given day was used; other entries were removed, because the last one presumably covered the previous. Fourth, some participants, of their own accord, provided reports after the third lab session; we removed these reports because they were not part of the study protocol. Additionally, for analysis of the full five-week experimental manipulation, we also excluded all data (including those pre-manipulation) of those who did not attend the second and third laboratory sessions, as well as all reports from both members of one couple because both members independently and privately reported at the third lab session that the expresser had revealed the GET instructions to the target after receiving them, when we had asked them not to. As reported in the main text, regardless of these occasional missing data, we had extremely high compliance with the protocol, and more than 7,000 observations from which to draw conclusions.

***Cleaning self-reported time spent with partner.*** Participants estimated hours and minutes in free-response format, leaving occasional text-based rather than numeric entries. This occurred in 91 cases, that is, 1.14% of the data received. We used the following rules to convert these few cases to numbers. First, we replaced letters “o” and “p” in the answers to “0”, because 0 is the number key that is visually similar to the letters and the closest to them on the keyboard. We then changed phrases “few minutes” and “all day” to 0 and 24 hours respectively, because the times are arguably the best numerical estimates of the meanings of the phrases. Finally, we deleted entries of “-”, “Z1”, “the whole time”, “the rest”, and any individual times exceeding 24 hours and left them as missing data, because their meanings were uncertain to unreasonable.

## Experimental manipulation

In the second laboratory session, members of the couple were randomly assigned to the roles of expresser and target, first participating in a videorecorded conversation in which the expresser thanked the target for something s/he did recently that the expresser appreciated. This general paradigm has been described elsewhere^4^; in this study, the expresser privately received an additional request about *how* to express gratitude during the conversation, for a different research question; that hypothesis focused on the lab-based interaction was not supported, as documented elsewhere^5^, and we do not consider these instructions in the present manuscript.

Here, our research question is about the effect of random assignment to condition focused on daily behavior change: gratitude expression treatment (GET) vs. no-treatment control. At the end of the second laboratory session, while expresser and target were separated to complete final questionnaires, the GET manipulation was administered via Qualtrics online survey software. Expressers who were randomly assigned to GET received the following prompts, whereas those in the control condition did not. Making the plan to express gratitude was presented in four screens through which the participant advanced at their own pace.

# **RESULTS**

| Table S1. Descriptive Statistics of Daily Physical Proximity | | | | | | | |
| --- | --- | --- | --- | --- | --- | --- | --- |
| Variable (min.) | Baseline phase | | |  | Experimental phase | | |
|  | Mean | SD by level | |  | Mean | SD by level | |
|  |  | Day | Couple |  |  | Day | Couple |
| Overall presence | 698 | 327 | 290 |  | 725 | 343 | 289 |
| Control | 705 | 330 | 280 |  | 698 | 354 | 292 |
| Gratitude expression | 691 | 322 | 303 |  | 752 | 329 | 285 |
| Sleeping | 327 | 165 | 153 |  | 331 | 166 | 150 |
| Control | 338 | 169 | 145 |  | 325 | 169 | 155 |
| Gratitude expression | 316 | 161 | 160 |  | 336 | 163 | 146 |
| Hanging out | 240 | 198 | 126 |  | 246 | 212 | 108 |
| Control | 238 | 204 | 113 |  | 235 | 212 | 97 |
| Gratitude expression | 240 | 191 | 139 |  | 256 | 213 | 116 |
| Share project | 43 | 72 | 49 |  | 44 | 77 | 55 |
| Control | 44 | 81 | 53 |  | 41 | 79 | 54 |
| Gratitude expression | 41 | 62 | 45 |  | 48 | 74 | 55 |
| Separate project | 103 | 125 | 77 |  | 104 | 114 | 95 |
| Control | 111 | 136 | 85 |  | 100 | 113 | 99 |
| Gratitude expression | 94 | 112 | 67 |  | 107 | 116 | 93 |
| Remote communication | 64 | 113 | 93 |  | 52 | 92 | 82 |
| Control | 70 | 122 | 104 |  | 59 | 98 | 89 |
| Gratitude expression | 58 | 102 | 82 |  | 45 | 85 | 76 |
| *Note:* All SDs are significantly larger than 0, showing meaningful interdependence between partners within a couple as well as fluctuations from one day to another. In contrast, many variance components, thus SDs, of the random slope of role are shown as superfluous, not significantly larger than 0, and shown instead in Table S2. As in the baseline models of shared time, this is likely caused by the fact that both partners in a couple were reporting on their “shared” time. | | | | | | | |

| Table S2. Supplementary Descriptive Statistics of Daily Physical Proximity | | |
| --- | --- | --- |
| Variable (min.) | SD of role random slope (partner discrepancy) | |
|  | Baseline phase | Experimental phase |
| Overall Time in Physical Proximity | 0.00 ^a^ | 19.91 |
| Sleeping | 0.00 ^a^ | 0.00 ^a^ |
| Hanging out | 0.00 ^a^ | 21.78 |
| Share project | 23.37 | 25.36 |
| Separate project | 37.73 | 33.92 |
| Remote communication | 40.79 | 31.89 |
| ^a^ denotes inestimable parameters automatically set to 0. | | |

| Table S3. Main Effect Statistics for the Models estimating GET Effects on Time Changes for Phase and Condition (supplement to manuscript Table 1 and Figure 2) | | | | | | | |
| --- | --- | --- | --- | --- | --- | --- | --- |
|  | Est. | SE | df | t | p | [ CI 95% ] | |
| Overall presence * | 709.82 | 25.49 | 123.36 | 27.85 | < .001 | 659.38 | 760.27 |
| Phase * | 16.00 | 5.82 | 1948.40 | 2.75 | .006 | 4.59 | 27.41 |
| Condition | 12.96 | 25.49 | 123.36 | 0.51 | .612 | −37.49 | 63.41 |
| Sleeping * | 328.51 | 13.31 | 123.07 | 24.68 | < .001 | 302.16 | 354.86 |
| Phase * | 1.41 | 2.61 | 2072.60 | 0.54 | .588 | −3.70 | 6.53 |
| Condition | −2.53 | 13.31 | 123.07 | −0.19 | .850 | −28.88 | 23.82 |
| Hanging out * | 242.34 | 9.96 | 124.57 | 24.32 | < .001 | 222.62 | 262.06 |
| Phase * | 4.46 | 3.36 | 2046.18 | 1.33 | .185 | −2.13 | 11.05 |
| Condition | 7.62 | 9.96 | 124.58 | 0.77 | .446 | −12.10 | 27.34 |
| Share project * | 43.44 | 4.70 | 123.78 | 9.23 | < .001 | 34.12 | 52.75 |
| Phase * | 1.31 | 1.05 | 2274.24 | 1.24 | .215 | −0.76 | 3.37 |
| Condition | 1.08 | 4.71 | 123.78 | 0.23 | .818 | −8.23 | 10.40 |
| Separate project * | 103.71 | 7.65 | 123.45 | 13.56 | < .001 | 88.58 | 118.85 |
| Phase * | 1.82 | 1.82 | 2004.88 | 1.00 | .317 | −1.74 | 5.38 |
| Condition | −3.54 | 7.65 | 123.45 | −0.46 | .645 | −18.67 | 11.60 |
| Remote communication | 57.05 | 7.61 | 122.80 | 7.49 | < .001 | 41.98 | 72.12 |
| Phase * | −5.42 | 1.62 | 1771.96 | −3.34 | .001 | −8.60 | −2.23 |
| Condition | −6.63 | 7.61 | 122.80 | −0.87 | .386 | −21.70 | 8.44 |
| * indicates p < .05; Manuscript Table 1 reports the overall two-way interaction between phase and condition on each outcome; for comprehensive reporting, this Table reports the additional statistics from those models, here with the first row of each domain of shared time representing the intercept of the model, and each inset row representing the main effects from those models. | | | | | | | |

| Table S4. GET Effects for Members of Couples Where the Expressers were of Different Sexes | | | | | | | | |
| --- | --- | --- | --- | --- | --- | --- | --- | --- |
|  | Est. | SE | df | t | p | | [ CI 95% ] | |
| Intercept * | 709.54 | 25.11 | 121.23 | 28.26 | .000 | 659.83 | | 759.25 |
| Phase (P) * | 16.03 | 5.82 | 1943.33 | 2.75 | .006 | 4.62 | | 27.45 |
| Condition (C) | 12.62 | 25.11 | 121.23 | 0.50 | .616 | −37.09 | | 62.33 |
| Expresser sex (S) * | −57.49 | 25.11 | 121.23 | −2.29 | .024 | −107.21 | | −7.78 |
| P × C * | 17.96 | 5.82 | 1943.33 | 3.09 | .002 | 6.55 | | 29.38 |
| C × S | 20.00 | 25.11 | 121.23 | 0.80 | .427 | −29.71 | | 69.71 |
| P × S | 5.49 | 5.82 | 1943.33 | 0.94 | .346 | −5.93 | | 16.91 |
| C × P × S | 0.14 | 5.82 | 1943.33 | 0.02 | .981 | −11.28 | | 11.55 |
| * indicates p < .05. | | | | | | | | |

Conclusion: There is no evidence that the GET effect varies with expressers’ sexes.

| Table S5. Moderation of Sex on the Associations Between rs6449182 and Baseline Daily Relationship Measures | | | | | | | | |
| --- | --- | --- | --- | --- | --- | --- | --- | --- |
|  | Est. | SE | df | t | p | OR | [ CI 95% ] | |
| Relationship satisfaction | 0.09 | 0.17 | 188.02 | 0.54 | .590 |  | −0.24 | 0.43 |
| Gratitude | 0.11 | 0.33 | 3090.00 | 0.33 | .744 | 1.11 | 0.59 | 2.11 |
| Love | 0.13 | 0.47 | 3090.00 | 0.28 | .782 | 1.14 | 0.45 | 2.86 |
| Relationship maintenance | 0.01 | 0.03 | 228.46 | 0.32 | .748 |  | −0.06 | 0.08 |
| Time spent (min.) | −7.78 | 37.70 | 703.38 | −0.21 | .837 |  | −81.80 | 66.25 |
| * indicates p < .05; Est. represents an unstandardized regression coefficient from a multi-level model regressing the dependent measure from the 14-night baseline phase on genotype. | | | | | | | | |

Conclusion: There is no evidence that the effects vary with participants’ sexes.

| Table S6. GET Effects for Individuals with Different rs6449182 Genotypes | | | | | | | |
| --- | --- | --- | --- | --- | --- | --- | --- |
|  | Est. | SE | df | t | p | [ CI 95% ] | |
| rs6449182 * | 710.00 | 25.97 | 119.26 | 27.34 | < .001 | 658.57 | 761.43 |
| Phase (P) * | 16.19 | 5.85 | 1920.70 | 2.77 | .006 | 4.72 | 27.67 |
| Condition (C) | 12.42 | 25.97 | 119.26 | 0.48 | .633 | −39.01 | 63.85 |
| rs6449182 | −25.65 | 25.23 | 119.89 | −1.02 | .311 | −75.60 | 24.30 |
| P × C * | 17.45 | 5.85 | 1920.70 | 2.98 | .003 | 5.98 | 28.92 |
| C × rs6449182 | 17.12 | 25.23 | 119.89 | 0.68 | .499 | −32.83 | 67.07 |
| P × rs6449182 | 1.14 | 5.80 | 1938.35 | 0.20 | .844 | −10.23 | 12.51 |
| C × P × rs6449182 * | −12.93 | 5.80 | 1938.35 | −2.23 | .026 | −24.30 | −1.56 |
| * indicates p < .05; rs6449182 uses additive-G coding; the first row represents the intercept of the model. | | | | | | | |

***Moderation by role.***

To ensure that the above findings were not solely driven by expressers’ nightly reports *biased by* their genotype, we fit an additional model in which role and its interactions with all other predictors were included as predictors.he focal moderation effect of rs6449182 in Table S4 (phase X condition x rs6449182) was not further moderated by role (est. = 0.63, SE = 5.76, df = 1987.58, t = 0.11, p = .913, [ CI 95% ] = [−10.67, 11.93]). Full model statistics not reported.

***Moderation by expresser/target sex.***

To ensure that the above findings were not solely driven by expressers of one but not the other sex (recall that the expression targets were always the opposite sex so expresser and target sex are fully correlated and statistically the same) we fit an additional model in which expresser sex and its interactions with all other predictors were included as predictors, and found that the focal moderation effect of rs6449182 in Table S6 (phase X condition x rs6449182) was not further moderated by expresser/target sex (est. = −5.95, SE = 5.86, df = 1914.22, t = −1.02, p = . 310, [ CI 95% ] = [−17.44, 5.54]). Full model statistics not reported.

***Moderation by ethnicity.***

In lieu of population stratification corrections to ensure that the above findings were not solely driven by our majority-White participants, we fit an additional model in which ethnicity (White coded 0; non-White coded 1) and its interactions with all other predictors were included as predictors, finding that the focal moderation effect of rs6449182 in Table S6 (phase X condition x rs6449182) was not further moderated by ethnicity in our sample. See Table S7, final row.

| Table S7. Testing for Manipulation Effects by Ethnicity | | | | | | | |  |
| --- | --- | --- | --- | --- | --- | --- | --- | --- |
|  | Est. | SE | df | t | p | [ CI 95% ] | | |
| rs6449182 * | 709.79 | 26.06 | 118.84 | 27.24 | < .001 | 658.19 | 761.38 | |
| Ethnicity (E) | 0.66 | 10.66 | 1426.09 | 0.06 | .951 | -20.25 | 21.57 | |
| Phase (P) * | 16.07 | 5.86 | 1908.73 | 2.74 | .006 | 4.57 | 27.57 | |
| × E | −1.03 | 5.86 | 1878.81 | -0.18 | .861 | -12.51 | 10.46 | |
| Condition (C) | 12.63 | 26.06 | 118.84 | 0.49 | .629 | -38.96 | 64.23 | |
| × E | −1.06 | 10.66 | 1426.09 | -0.10 | .921 | -21.97 | 19.85 | |
| rs6449182 | −25.56 | 25.35 | 120.08 | -1.01 | .315 | -75.74 | 24.64 | |
| × E | 0.68 | 10.26 | 1473.52 | 0.07 | .947 | -19.44 | 20.79 | |
| P × C * | 17.55 | 5.86 | 1908.73 | 2.99 | .003 | 6.02 | 29.02 | |
| × E | 9.52 | 5.86 | 1878.81 | 1.63 | .104 | -1.97 | 21.01 | |
| C × rs6449182 | 17.04 | 25.35 | 120.08 | 0.67 | .503 | -33.14 | 67.22 | |
| × E | 2.82 | 10.26 | 1473.52 | 0.28 | .783 | -17.30 | 22.94 | |
| P × rs6449182 | 2.49 | 5.86 | 1914.74 | 0.43 | .671 | -9.00 | 13.98 | |
| × E | 7.86 | 6.13 | 1873.10 | 1.28 | .200 | -4.16 | 19.87 | |
| C × P × rs6449182 * | −13.62 | 5.86 | 1914.74 | -2.33 | .020 | -25.10 | -2.13 | |
| × E | −6.41 | 6.13 | 1873.10 | -1.05 | .295 | -18.43 | 5.60 | |
| * indicates p < .05; rs6449182 uses the additive-G coding; ethnicity uses a white (0) v. non-white (1) coding and then standardized; the first row of each snp is for the intercept of the model. | | | | | | | |  |

Conclusion: There is no evidence that the effects are driven by White participants.

**Considering Relationship Satisfaction as an Outcome**

**Background:** Spending time co-present with a partner may reflect a relational *bond*, by which we mean commitment to or investment into the relationship^6^. However, a different outcome that has been of interest in the broader literature on gratitude in relationships has been an evaluation of the *quality* of a relationship, often labeled relationship satisfaction^7, 8^. Satisfaction with the relationship is an evaluation of the costs and rewards in the relationship, and is distinct from commitment to the relationship^9^. Although not the focus of the present investigation, here we present a test of whether the manipulation influenced relationship satisfaction, to contribute to that extant literature.

| Table S8. GET Effects on Relationship Satisfaction for Phase and Condition | | | | | | | |
| --- | --- | --- | --- | --- | --- | --- | --- |
|  | Est. | SE | df | t | p | [ CI 95% ] | |
| Relationship satisfaction * | 7.47 | 0.07 | 122.46 | 106.71 | .000 | 7.33 | 7.61 |
| Phase (P) * | 0.05 | 0.02 | 1972.77 | 2.93 | .003 | 0.02 | 0.08 |
| Condition (C) | 0.03 | 0.07 | 122.46 | 0.37 | .715 | −0.11 | 0.16 |
| P × C | −0.01 | 0.02 | 1972.77 | −0.83 | .409 | −0.05 | 0.02 |
| * indicates p < .05; the first row represents the intercept of the model. | | | | | | | |

Conclusion: There is no evidence that GET, compared to the control, increased relationship satisfaction. Instead, both conditions increased significantly and statistically indistinguishably, over time.

It is possible that, consistent with prior research^7^, increasing the frequency of expressed gratitude in ongoing relationships does not increase relationship satisfaction. In future research with this outcome, it will be worth considering the theoretical and methodological context of these results when designing the study. For example, because these were very satisfied couples at baseline (*M* = 7.42 on a 1-to-9 scale, across all participants and days), the brief manipulation may not have been powerful enough to overcome potential ceiling effects of the measure, especially after taking into account the ostensible effect of the visit to the lab that is reflected in the significant effect of study phase (see Table S8); it is possible that somewhat less satisfied couples or use of a different scale that better differentiates at the upper end of the continuum would mitigate such an effect in the future.

# **EXPLORATORY RESULTS FOR rs3796863**

## Background

As described in the main manuscript, we focused on rs6449182 due to its prior significant associations related to expressed gratitude in daily life^10^. However, we genotyped for one other SNP, also in the *CD38* gene, rs3796863, which was not significantly associated with daily or observed expressed gratitude in our prior study^10^ but has been the target of greater – and increasing -- psychological study than rs6449182. For the sake of thoroughness and transparency, we explored associations with this SNP and report the results here.

In contrast to rs6449182, which is located in intron 1, rs3796863 is located at the other end of the gene in intron 7. With respect to neural function, rs3796863 has been associated with levels of unextracted plasma oxytocin^11^ and alcohol stimulated dopamine release^12^, suggesting that rs3796863 is a functional SNP. Regarding social behavior and partner perceptions in the context of ongoing relationships, three papers using correlational approaches provide mixed evidence of associations between rs3796863 and overall relationship well-being (e.g., satisfaction^10, 13, 14^), with the majority of the statistically significant findings suggesting individuals with the CC genotype (vs. carriers of the A Allele [i.e. AC and AA genotypes grouped together]) experienced more bonding-relevant cognitions about their relationship partner (e.g., perceived partner responsiveness^10^, trust^13^). Additionally, of relevance to the concept of partner preference^15^, a recent experiment showed that a partner preference task worked as predicted for individuals who carried the A allele (vs. the CC genotype); for example, A-allele infants preferred to look at an image of a person who had previously smiled at them, compared to the looking preferences of the CC individuals^16^. In sum, despite our prior null results, there is ample reason to explore this SNP as it relates to the present hypotheses.

## Genotyping

Salivary DNA collection and extraction procedures as well as genotyping procedures replicated those in our prior research^10^. Similarly, the rs3796863 SNP was coded in a manner to be consistent with prior work, which has shown that the A allele appears to function in a dominant manner (CC = 0, *n* = 101; AC or AA = 1, *n* = 144). The genotyping call rate was 100% and the genotyping results conformed to Hardy-Weinberg equilibrium (*X^2^* = 0.29, *df*  = 1, *p* = 0.58).

## Results

| Table S9. Associations Between rs3796863 and Baseline Daily Relationship Measures | | | | | | | | |
| --- | --- | --- | --- | --- | --- | --- | --- | --- |
|  | Est. | SE | df | t | p | OR | [ CI 95% ] | |
| Relationship satisfaction | 0.11 | 0.11 | 199.30 | 1.00 | .320 |  | −0.10 | 0.32 |
| Gratitude † | 0.37 | 0.22 | 3107.00 | 1.74 | .083 | 1.45 | 0.95 | 2.22 |
| Love | 0.14 | 0.32 | 3107.00 | 0.44 | .658 | 1.15 | 0.62 | 2.13 |
| Relationship maintenance | 0.01 | 0.02 | 239.09 | 0.69 | .492 |  | −0.03 | 0.06 |
| Time spent (min.) | −19.27 | 23.94 | 722.35 | −0.81 | .421 |  | −66.29 | 27.74 |
| † indicates p < .10 | | | | | | | | |

Consistent with Algoe & Way (2014), rs3796863 was not significantly associated with any of the five outcomes, though was approaching significance in its association with expressed gratitude.

| Table S10. Manipulation Effects for Individuals with Different rs3796863 Genotypes | | | | | | | |
| --- | --- | --- | --- | --- | --- | --- | --- |
|  | Est. | SE | df | t | p | [ CI 95% ] | |
| rs3796863 * | 715.89 | 26.23 | 119.36 | 27.30 | < .001 | 663.96 | 767.82 |
| Phase (P) * | 18.94 | 6.00 | 1917.18 | 3.16 | .002 | 7.18 | 30.71 |
| Condition (C) | 17.18 | 26.23 | 119.36 | 0.66 | .514 | −34.75 | 69.11 |
| rs3796863 | 4.78 | 26.26 | 119.41 | 0.18 | .856 | −47.22 | 56.78 |
| P × C * | 18.42 | 6.00 | 1917.18 | 3.07 | .002 | 6.66 | 30.19 |
| C × rs3796863 | 5.43 | 26.26 | 119.41 | 0.21 | .836 | −46.57 | 57.44 |
| P × rs3796863 | 4.71 | 6.00 | 1913.06 | 0.78 | .433 | −7.07 | 16.48 |
| C × P × rs3796863* | 12.33 | 6.00 | 1913.06 | 2.05 | .040 | 0.56 | 24.11 |
| * indicates p < .05; rs3796863 used the dominant-A coding; the first row is for the intercept of the model. | | | | | | | |

The results of the three-way interaction reveal that couples whose expressers were A-carriers (coded 1) showed greater increases in overall time spent in the physical presence of the partner, relative to couples whose expressers were CC (coded 0), as a result of the experimental manipulation. All statistics from this full model can be found in Table S10, above.

Results in Table S11 show that the manipulation only affected the couples in which the expresser was an A-carrier of rs3796863, but did not influence couples in which the expresser was a CC individual. Analogous to findings for rs6449182, rs3796863 A-carriers comprise 58.7% (i.e., the majority) of the expresser sample, and these were the individuals most likely to express gratitude during the baseline phase (see Table S9).

| Table S11. Manipulation Effects on Overall Time Spent within Different Expresser rs3796863 Genotypes | | | | | | | |
| --- | --- | --- | --- | --- | --- | --- | --- |
|  | Est. | SE | df | t | p | [ CI 95% ] | |
| rs3796863 |  |  |  |  |  |  |  |
| CC | 15.62 | 37.14 | 1902.46 | 0.42 | .674 | −57.21 | 88.45 |
| A Allele Carriers* | 115.59 | 31.45 | 1927.96 | 3.68 | < .001 | 53.92 | 177.27 |
| * indicates p < .05. Each row presents the results of the simple effects (i.e., two-way interaction between experimental condition and phase) within each expresser genotype of a given snp. | | | | | | | |

Breaking down the significant two-way interaction for A-carriers, the simple effects within condition show that individuals in GET significantly increased in the overall time they shared with the partners (est. = 84.60, SE = 24.33, df = 1913.06, t = 3.48 p = .001, [ CI 95% ] = [36.88, 132.31]), while those in the control condition did not change (est. = -30.99, SE = 24.33, df = 1913.06, t = -1.27, p = .203, [ CI 95% ] = [-78.71, 16.72]).

***Moderation by role.***

To ensure that the findings were not solely driven by expressers’ nightly reports *biased by* their genotype, we fit a model in which role and its interactions with all other predictors were included as predictors, and found that the that the focal moderation effect of rs3796863 in Table S10 (phase X condition x rs3796863) was not further moderated by role (est. = −4.30, SE = 5.95, df = 1866.29, t = −0.72, p = .474, [ CI 95% ] = [−15.92, 7.40]). Full model statistics not reported.

***Moderation by ethnicity.***

Finally, we tested whether this focal moderation effect of rs3796863 in Table S10 (phase X condition x rs3796863) was further moderated by ethnicity (coded White = 1, all other racial/ethnic demographics = 0); it was not. Thus, there is no evidence that the effects are driven by White participants. See Table S12, final row.

| Table S12. Testing for Manipulation Effects by Ethnicity with rs3796863 | | | | | | | |
| --- | --- | --- | --- | --- | --- | --- | --- |
|  | Est. | SE | df | t | p | [ CI 95% ] | |
| rs3796863 * | 715.89 | 26.31 | 119.05 | 27.21 | < .001 | 663.79 | 767.99 |
| Ethnicity (E) | 1.90 | 11.50 | 1341.46 | 0.17 | .868 | -20.66 | 24.47 |
| Phase (P) * | 19.59 | 6.05 | 1905.36 | 3.24 | .001 | 7.73 | 31.47 |
| × E | −0.86 | 6.02 | 1860.00 | -0.14 | .887 | -12.67 | 10.95 |
| Condition (C) | 17.18 | 26.31 | 119.05 | 0.65 | .515 | -34.92 | 69.28 |
| × E | 0.19 | 11.50 | 1341.46 | 0.02 | .987 | -22.38 | 22.76 |
| rs3796863 | 4.29 | 26.35 | 119.08 | 0.16 | .871 | -47.88 | 56.46 |
| × E | −0.36 | 11.09 | 1380.21 | -0.33 | .974 | -22.12 | 21.40 |
| P × C * | 18.64 | 6.05 | 1905.36 | 3.08 | .002 | 6.77 | 30.51 |
| × E | 6.29 | 6.02 | 1857.00 | 1.05 | .296 | -5.51 | 18.10 |
| C × rs3796863 | 5.60 | 26.35 | 119.08 | 0.21 | .832 | -46.57 | 57.77 |
| × E | 0.03 | 11.09 | 1380.22 | 0.00 | .998 | -21.73 | 21.79 |
| P × rs3796863 | 4.05 | 6.05 | 1898.29 | 0.67 | .505 | -7.84 | 15.91 |
| × E † | −10.50 | 6.10 | 1844.49 | -1.72 | .086 | -22.47 | 1.47 |
| C × P × rs3796863 * | 11.97 | 6.06 | 1898.29 | 1.98 | .048 | 0.10 | 23.85 |
| × E | 4.04 | 6.10 | 1844.49 | 0.66 | .508 | -7.93 | 16.01 |
| * and † indicates p < .05 and .10 respectively; ethnicity uses a white (0) v. non-white (1) coding and then standardized; the first row is for the intercept of the model. | | | | | | | |

## rs3796863 and Gratitude: Comment

Overall, paired with our primary analyses focused on rs6449182, *CD38* genotype, as indexed by two different SNPs, modulated the impact of a bonding manipulation on subsequent time spent in the physical presence of the romantic partner. Importantly, although the key finding of condition on change in time spent was modulated by rs3796863, the effective genotype (A allele carriers) represented the majority of expressers in our sample (58.7%); that is, consistent with the broad literature on expressed gratitude, most people have this capacity.

Regarding main effects (Table S9), although the present pattern of findings is consistent with our prior paper (which produced only 1 of 10 statistically significant main effects for rs3796863) which focused on a variety of bonding-relevant behaviors and self-reported experiences, we note that two recent papers do show significant associations between rs3796863 and a variety of conceptually related variables (e.g., “communal behavior”, trait gratitude, and relationship satisfaction^13, 14^). This is promising, yet requires clarification of two important points that we and those authors have emphasized: (1) there is vast theoretical diversity within relationship-relevant outcomes (e.g., between spending time with the partner and expressing gratitude; or between low conflict, providing support during negative experiences, and expressing gratitude^17, 18, 19, 20, 21, 22^), (2) even two variables that sound similar can tap meaningfully different psychological phenomena (e.g., trait gratitude, about individual differences in the regular *experience* of gratitude, versus expressed gratitude, a social behavior directed toward another individual). Additionally, we note that, in contrast to the direct replication of the association between rs6449182 and daily expressed gratitude from Algoe & Way (2014) in the present study, both the present and prior study showed associations between rs3796863 and daily expressed gratitude that were just trending toward statistical significance by the conventional standard of *p* < .05 (p = .09 in both cases). However, the rs3796863 associations were trending in opposite directions in the two studies. Altogether, the very promising, though somewhat inconsistent, pattern of associations for rs3796863 across now four studies of individuals in close relationships is well worth future research in samples employing the same measures that were used in all four prior studies to test for direct replication and identifying potential moderators of the genetic effects.

# **References**

1. Gable SL, Reis HT, Downey G. He Said, She Said:A Quasi-Signal Detection Analysis of Daily Interactions Between Close Relationship Partners. *Psychological Science* **14**, 100-105 (2003).

2. Algoe S, Fredrickson B. Carolina Couples Study, 2008 ("CC08").). V1 edn. UNC Dataverse (2019).

3. Kubacka KE, Finkenauer C, Rusbult CE, Keijsers L. Maintaining Close Relationships:Gratitude as a Motivator and a Detector of Maintenance Behavior. *Personality and Social Psychology Bulletin* **37**, 1362-1375 (2011).

4. Algoe SB, Fredrickson BL, Gable SL. The social functions of the emotion of gratitude via expression. *Emotion* **13**, 605-609 (2013).

5. Algoe SB, Kurtz LE, Hilaire NM. Putting the “You” in “Thank You”:Examining Other-Praising Behavior as the Active Relational Ingredient in Expressed Gratitude. *Social Psychological and Personality Science* **7**, 658-666 (2016).

6. Huxhold O, Fiori KL, Windsor T. Rethinking Social Relationships in Adulthood: The Differential Investment of Resources Model. *Personality and Social Psychology Review* **26**, 57-82 (2022).

7. Algoe SB, Zhaoyang R. Positive psychology in context: Effects of expressing gratitude in ongoing relationships depend on perceptions of enactor responsiveness. *The Journal of Positive Psychology* **11**, 399-415 (2016).

8. Gordon AM, Impett EA, Kogan A, Oveis C, Keltner D. To have and to hold: Gratitude promotes relationship maintenance in intimate bonds. *Journal of Personality and Social Psychology* **103**, 257-274 (2012).

9. Rusbult CE. A longitudinal test of the investment model: The development (and deterioration) of satisfaction and commitment in heterosexual involvements. *Journal of Personality and Social Psychology* **45**, 101-117 (1983).

10. Algoe SB, Way BM. Evidence for a role of the oxytocin system, indexed by genetic variation in CD38, in the social bonding effects of expressed gratitude. *Social Cognitive and Affective Neuroscience* **9**, 1855-1861 (2014).

11. Feldman R. Oxytocin and social affiliation in humans. *Hormones and Behavior* **61**, 380-391 (2012).

12. Lee MR*, et al.* A role for the CD38 rs3796863 polymorphism in alcohol and monetary reward: evidence from CD38 knockout mice and alcohol self-administration, [11C]-raclopride binding, and functional MRI in humans. *The American Journal of Drug and Alcohol Abuse* **46**, 167-179 (2020).

13. Makhanova A, McNulty JK, Eckel LA, Nikonova L, Bartz JA, Hammock EAD. CD38 is associated with bonding-relevant cognitions and relationship satisfaction over the first 3 years of marriage. *Scientific Reports* **11**, 2965 (2021).

14. Sadikaj G, Moskowitz DS, Zuroff DC, Bartz JA. CD38 is associated with communal behavior, partner perceptions, affect and relationship adjustment in romantic relationships. *Scientific Reports* **10**, 12926 (2020).

15. Williams JR, Carter CS, Insel T. Partner Preference Development in Female Prairie Voles Is Facilitated by Mating or the Central Infusion of Oxytocina. *Annals of the New York Academy of Sciences* **652**, 487-489 (1992).

16. Krol KM, Monakhov M, Lai PS, Ebstein RP, Grossmann T. Genetic variation in CD38 and breastfeeding experience interact to impact infants’ attention to social eye cues. *Proceedings of the National Academy of Sciences* **112**, E5434-E5442 (2015).

17. Algoe S, Jolink TA. Social Bonds: A New Look at an Old Topic. In: *Social Psychology: Handbook of Basic Principles* (eds Lange PAMV, Higgins ET, Kruglanski AW). Guilford Press (2020).

18. Algoe SB. Positive Interpersonal Processes. *Current Directions in Psychological Science* **28**, 183-188 (2019).

19. Gable SL, Gonzaga GC, Strachman A. Will you be there for me when things go right? Supportive responses to positive event disclosures. *Journal of Personality and Social Psychology* **91**, 904-917 (2006).

20. Gable SL, Gosnell CL, Maisel NC, Strachman A. Safely testing the alarm: Close others' responses to personal positive events. *Journal of Personality and Social Psychology* **103**, 963-981 (2012).

21. Gable SL, Reis HT. Appetitive and aversive social interaction. In: *Close romantic relationships: Maintenance and enhancement.*). Lawrence Erlbaum Associates Publishers (2001).

22. Rusbult CE, Martz JM, Agnew CR. The Investment Model Scale: Measuring commitment level, satisfaction level, quality of alternatives, and investment size. *Personal Relationships* **5**, 357-391 (1998).
